# Mushroom Body Specific Transcriptome Analysis Reveals Dynamic Regulation of Learning and Memory Genes After Acquisition of Long-Term Courtship Memory in *Drosophila*

**DOI:** 10.1534/g3.118.200560

**Published:** 2018-08-29

**Authors:** Spencer G. Jones, Kevin C. J. Nixon, Melissa C. Chubak, Jamie M. Kramer

**Affiliations:** * Department of Neuroscience; † Department of Biology; ‡ Department of Physiology and Pharmacology; § Children’s Health Research Institute, University of Western Ontario, London, Ontario Canada

**Keywords:** long-term memory, transcriptome analysis, courtship conditioning

## Abstract

The formation and recall of long-term memory (LTM) requires neuron activity-induced gene expression. Transcriptome analysis has been used to identify genes that have altered expression after memory acquisition, however, we still have an incomplete picture of the transcriptional changes that are required for LTM formation. The complex spatial and temporal dynamics of memory formation creates significant challenges in defining memory-relevant gene expression changes. The *Drosophila* mushroom body (MB) is a signaling hub in the insect brain that integrates sensory information to form memories across several different experimental memory paradigms. Here, we performed transcriptome analysis in the MB at two time points after the acquisition of LTM: 1 hr and 24 hr. The MB transcriptome was compared to biologically paired whole head (WH) transcriptomes. In both, we identified more transcript level changes at 1 hr after memory acquisition (WH = 322, MB = 302) than at 24 hr (WH = 23, MB = 20). WH samples showed downregulation of developmental genes and upregulation of sensory response genes. In contrast, MB samples showed vastly different changes in transcripts involved in biological processes that are specifically related to LTM. MB-downregulated genes were highly enriched for metabolic function. MB-upregulated genes were highly enriched for known learning and memory processes, including calcium-mediated neurotransmitter release and cAMP signaling. The neuron activity inducible genes *Hr38* and *sr* were also specifically induced in the MB. These results highlight the importance of sampling time and cell type in capturing biologically relevant transcript level changes involved in learning and memory. Our data suggests that MB cells transiently upregulate known memory-related pathways after memory acquisition and provides a critical frame of reference for further investigation into the role of MB-specific gene regulation in memory.

Learning and memory can be measured in experimental organisms by observing altered behavior in response to manipulated experiences. The duration of behavioral changes induced by different learning and memory paradigms may be transient or stable (Tully *et al.* 2003; Hawkins *et al.* 2006). While the formation of both short-term and long-term memories require similar underlying molecular mechanisms such as calcium- and cAMP-dependent signaling pathways, only long-term memory (LTM) requires gene transcription and *de novo* protein synthesis (Brunelli *et al.* 1976; Montarolo *et al.* 1986; Lee 2015). Many genes have been implicated in LTM formation (Bliim *et al.* 2016), however, we still know very little about the spatial and temporal dynamics of gene regulation that are required for LTM.

The fruit fly, *Drosophila melanogaster*, has been a powerful model for the discovery of genes and molecular mechanisms underlying learning and memory (Livingstone *et al.* 1984; Dudai *et al.* 1976; Lee 2015). Transcriptome analysis has been used to identify gene expression changes in flies after the acquisition of LTM (Dubnau *et al.* 2003; Winbush *et al.* 2012; Bozler *et al.* 2017; Crocker *et al.* 2016; Widmer *et al.* 2018). Several studies have profiled transcript levels in whole heads after memory acquisition (Dubnau *et al.* 2003; Winbush *et al.* 2012; Bozler *et al.* 2017), which has led to the identification of genes that are required for LTM (Dubnau *et al.* 2003; Bozler *et al.* 2017). Despite the success of these whole head studies, LTM requires only a subset of neurons that are both spatially and temporally regulated (Cognigni *et al.* 2018; Pavlowsky *et al.* 2018; Zhao *et al.* 2018). As such, cell-type specific analysis of different neuronal subsets involved in LTM will be required to fully elucidate the molecular mechanisms of memory (Johnson *et al.* 2013).

The mushroom body (MB) is a region of the fly brain that is critical for normal memory (de Belle and Heisenberg 1994; McBride *et al.* 1999). This synaptically dense structure appears as a pair of neuropils each consisting of ∼2000 neurons with three distinct neuronal subtypes (α/β, α’/β’, and γ) that contribute the formation of 5 distinct lobes α, α’, β, β’, and γ (Lee and Luo 1999). Intrinsic MB neurons, called Kenyon cells (KC), form a hub for integration of sensory information from over 200 olfactory projection neurons, and 20 different types of modulatory dopaminergic neurons (DANs) (Aso *et al.* 2014). Projection neurons relay olfactory information and synapse with dendrites of the MB neurons in the calyx (Jefferis *et al.* 2007). DANs synapse on different locations along the axonal MB lobes and correspond to the location of 21 types of MB output neurons (MBON), converging to create a highly structured DAN-KC-MBON compartment (Aso *et al.* 2014; Cognigni *et al.* 2018). The coincident activation of DANs and MBONs is thought to be essential in eliciting a behavioral response to conditioning (Cognigni *et al.* 2018). Different memory paradigms, including olfactory appetitive and aversive conditioning, are known to require distinct, specialized input and output neuron types to produce the corresponding behavioral changes (Liu *et al.* 2012; Jefferis *et al.* 2007; Cognigni *et al.* 2018; Kirkhart and Scott 2015; Owald *et al.* 2015; Perisse *et al.* 2016). However, the requirement for KCs is consistent across many different types of memory (de Belle and Heisenberg 1994; McBride *et al.* 1999). For olfactory memory, it is known that γ KCs are required for STM, α/β KCs play a role in LTM, and α’/β’ in memory consolidation (Blum *et al.* 2009; Tomchik and Davis 2009; Krashes *et al.* 2007; Trannoy *et al.* 2011; Yu *et al.* 2006). Lobe requirements for courtship conditioning, the assay used in this study, are less well understood and have predominantly been tested during STM. However, it is thought that all lobes play some role in courtship memory, with a known circuit of neurons involving the γ lobe being required for early courtship memory formation (Montague and Baker 2016; Keleman *et al.* 2012; Zhao *et al.* 2018; McBride *et al.* 1999).

Due to its essential role in several forms of memory, the MB is a logical starting point in the search for LTM-dependent gene expression changes. MB-specific transcriptome analysis has led to the discovery of additional genes that are important for LTM (Crocker *et al.* 2016; Widmer *et al.* 2018). Crocker *et al.* used patch-clamp pipets to harvest RNA from specific sets of intrinsic and extrinsic MB neurons 30 min after olfactory avoidance training, revealing a novel role for light-sensing genes in a specific set of MBONs (Crocker *et al.* 2016). Widmer *et al.* used targeted DamID (TaDa) to profile RNA polymerase II (polII) binding in intrinsic MB neurons during four 12 hr time windows after memory acquisition (Widmer *et al.* 2018). This study identified differential polII binding for dozens of genes in each time window. Ten novel genes that are important for LTM were identified by RNAi screening of top candidates that showed differential polII binding 12-72 hr after memory acquisition (Widmer *et al.* 2018). These studies illustrate the potential of MB-specific transcriptome analysis in revealing novel memory genes. However, the dynamics of gene regulatory changes that occur in the MB after memory acquisition are still not well understood. In the MB, different transcription factors and chromatin modifiers are required for olfactory memory at different temporal stages of memory formation maintenance, but the global effect of these transcription factors is not known (Hirano *et al.* 2016). Clearly, there is still a lot to learn about the spatial and temporal regulation of gene expression that is required for long term memory.

Courtship conditioning is a well-established learning and memory paradigm that has been commonly used to investigate the molecular mechanisms underlying memory (McBride *et al.* 1999; Koemans *et al.* 2017; Kramer *et al.* 2011; Keleman *et al.* 2007). Courtship conditioning relies on male courtship behavior being modifiable in response to sexual rejection from a mated unreceptive female (Spieth 1974; Siegel and Hall 1979). After experiencing sexual rejection males show reduced courting attempts with other pre-mated females; an effect which can persist for several days (McBride *et al.* 1999; Keleman *et al.* 2007). Courtship memory forms via an enhanced behavioral response to the pheromone *cis*-vaccenyl-acetate (cVA), which is deposited on females by males during prior mating attempts (Keleman *et al.* 2012). The MB is required for the acquisition of normal long-term courtship memory (McBride *et al.* 1999). While courtship conditioning has molecular properties similar to other memory paradigms (Montague and Baker 2016), it is distinct in that it manipulates a complex, naturally occurring behavior with minimal experimental interference (Ejima *et al.* 2007; Keleman *et al.* 2012; Montague and Baker 2016). This makes courtship conditioning an attractive model that takes advantage of a robust but ethological form of memory.

Here, we contribute to the emerging picture of LTM-dependent gene regulation by using INTACT (isolation of nuclei tagged in a specific cell type) (Henry *et al.* 2012) to profile transcript level changes in MBs at two time points after the acquisition of long-term courtship memory. We find a dynamic effect on the regulation of learning and memory genes during LTM formation in MBs. Many known learning and memory genes are transiently upregulated in MBs one-hour after memory acquisition and return to baseline levels after 24 hr. This effect is specific to MBs, as whole head transcriptome analysis did not reveal gene regulatory changes in known memory associated biological pathways. This suggests a high demand for classic learning and memory genes in MBs after the acquisition of courtship memory and highlights the importance of sampling time and cell type in the detection of biologically relevant transcript level changes underlying memory.

## Materials and Methods

### Fly strains and culture

All *Drosophila melanogaster* strains were cultured at 25° and 70% humidity on a 12:12 light-dark cycle. Cultures were raised on a standard medium (cornmeal-sucrose-yeast-agar) supplemented by the mold inhibitors methyl-paraben and propanoic acid (Koemans *et al.* 2017). *R14H06-GAL4* flies were generated by the Janelia Farm Flylight project (Jenett *et al.* 2012) and obtained from Bloomington stock center (Stock #48667) and *UAS-unc84*::*GFP* flies were donated by Gilbert L. Henry (Henry *et al.* 2012). For courtship conditioning assays and transcriptome analysis heterozygotes were generated by crossing *UAS-unc84*::*GFP*; *R14H06-GAL4* flies to *P{CaryP}attP2* (Bloomington stock# 36303). The resulting progeny referred to as MB-unc84 have the genotype *UAS-unc84*::*GFP*/+;*R14H06-GAL4*/*attP2*. Females used in courtship conditioning were a mixed Canton-S/Oregon-R genetic background generated by J.M. Kramer.

### Courtship conditioning and sample collection

Long-term courtship memory was induced as described (Koemans *et al.* 2017). Newly eclosed *MB-unc84* males were collected and individually held in an isolation chamber for four to six days. Males were then trained by introducing a single pre-mated female into the isolation chamber for a period of seven hours. After training, males were separated from females and kept in isolation. Flies being used for RNA-seq analysis were collected one-hour after sexual rejection (1h-AR) and 24-hours after rejection (24h-AR). Naïve flies were also collected, and all flies were collected and flash frozen at the same time of day to avoid any gene regulatory effects due to circadian rhythm. Fly heads were isolated from the abdomen, wings, and legs by vortexing followed quickly by separation through a series of sieves. Heads were then stored at -80° for future processing by INTACT. For each day of courtship conditioning when flies were collected for transcriptome analysis, a subset of naïve and trained males were tested for LTM induction. Statistical significance of courtship suppression was evaluated using a Mann-Whitney *U*-test.

### Isolation of nuclei tagged in a specific cell-type (INTACT)

MB specific transcriptome analysis was accomplished using a described INTACT protocol with several modifications (Henry *et al.* 2012). Fly heads were suspended in 1 ml of homogenization buffer (25 mM KCl, 5 mM MgCl2, 20 mM tricine, 0.15 mM spermine, 0.5 mM spermidine, 10 mM β-glycerophosphate, 0.25 mM sucrose, RNAsin Plus RNase Inhibitors (Fisher Scientific: PRN2615), 1X Halt protease inhibitors (Thermo Fisher Scientific: 78430), pH 7.8) and ground with a pestle. To disrupt the cell membrane and release nuclei into solution NP40 was added to the homogenate to an end concentration of 0.3% and the solution was Dounce homogenized 6 times using the tight pestle. The 1 ml nuclear extract was passed through a 40 μm cell strainer and a 50 μl input sample was removed. This input fraction is representative of the whole head, containing both MB-specific GFP nuclei and untagged non-MB nuclei. Input fractions were centrifuged to obtain a nuclear pellet which would later be used as a source for whole head RNA sequencing.

Antibody-bound magnetic beads were freshly prepared for each immunopurification by absorbing 1μg of anti-GFP antibody (Invitrogen: G10362) to 60 μl of Protein G Dynabeads (Invitrogen: 10004D) according to the manufacturer’s instructions. To reduce non-specific binding nuclear extracts were pre-cleared by adding 60 μl of beads with no anti-GFP antibody. GFP labeled nuclei were then immunoprecipitated using GFP bound beads for 30 min at 4° with rotation. After washing, these remaining bead-bound nuclei represented the MB-specific fraction that was directly processed for RNA-sequencing.

To investigate the specificity of this protocol we calculated the proportion of INTACT (MB) and input (WH) nuclei with GFP for three independent replicates. Nuclei were labeled with 20mM DRAQ5 (abcam: ab108410) at room temperature for 30 min. Several slides were prepared for each sample and 3 fields of view were captured for each slide using a Zeiss AxioImager Z1 microscope. The average percentage of GFP nuclei was calculated for each biological replicate by manually counting nuclei. The total number of nuclei in WH and MB samples was not significantly different (*P* = 0.19, Student’s *t*-test), but the percentage of GFP positive nuclei was drastically different (**see results**).

### Adult brain dissection, staining and confocal microscopy

To observe the expression domain of *R14H06-GAL4* in adult brains we crossed this line to *UAS-mCD8-GFP* (Bloomington stock # 5137) and *UAS-unc84*::*GFP* and performed confocal microscopy. Brains were dissected in PBS and fixed with 4% paraformaldehyde for 45 min at room temperature. Counterstaining was performed with nc82 primary antibodies (1:50 dilution – developmental studies hybridoma bank) and DyLight 594 secondary antibodies (1:400 dilution). Brains were mounted in Vectashield (Vector Laboratories) and imaged using a Zeiss LSM 510 duo vario confocal microscope. Confocal projections were captured with 1 μm slices and processed using Image J software (Fiji) and Adobe Photoshop (Schindelin *et al.* 2012).

### RNA isolation and RNA-sequencing

RNA was isolated using a PicoPure RNA Isolation Kit (Invitrogen: KIT0204) for both the WH and MB fractions according to the manufacturer instructions. Sequencing libraries were prepared using the Nugen Ovation Drosophila RNA-Seq System 1-16 (Nugen: NU035032) kit according to instructions. cDNA was sheared to a target size between 200-300 bp using a Covaris S2 sonicator according to the manufacturer’s protocol. Library size was verified using the Agilent Bioanalyzer High Sensitivity DNA Kit and quantified using a Q-bit fluorometer. Libraries were sequenced on an Illumina NextSeq500 using the high output v2 75 cycle kit to a read length of 75 bp with single-end reads at London Regional Genomics Centre.

### RNA-seq data analysis

Raw sequence reads were trimmed using Prinseq (version 0.20.4) quality trimming to a minimum base quality score of 30 (error probability of 1 in 1,000 base calls) (Schmieder and Edwards 2011). Trimmed reads were then aligned to the *D. melanogaster* genome (Ensembl release 88, dm6) using STAR (version 2.5.3a) (Dobin *et al.* 2013; Aken *et al.* 2016). To ensure mushroom body specificity of MB samples compared to WH samples, we also aligned reads to the *C. elegans* unc-84 gene (NC_003284.9). Only uniquely aligned reads with a maximum of four mismatches were used for downstream analysis. Gene counts were obtained using HTSeq-count (version 0.7.1) using the default union settings to generate genic regions (Anders *et al.* 2015). To identify differentially expressed (DE) genes we used DESeq2 (version 1.18.1) (Love *et al.* 2014). Cut-off requirements for a gene to be called as DE were q < 0.05 and fold change > 1.3 up or down. Genes mapped to the Y chromosome were removed from the final DE lists. Principal component analysis (PCA) was performed for all samples together, as well as WH and MB samples separately, using the *plotPCA* function within DESeq2 (Figure S1 A-C). To identify groups of genes with similar trends of transcriptional regulation in response to courtship conditioning we used the ‘stats’ package in R (version 3.3.3) to perform *k*-means clustering on log_2_ fold changes (Love *et al.* 2014; R Core Team 2016).

### GO analysis

Gene ontology (GO) analysis was performed using PANTHER (version 13.1) (Ashburner *et al.* 2000; The Gene Ontology Consortium 2017; Mi *et al.* 2017). For GO analysis for biological processes of DE genes between MB and WH samples we included all terms with a *P* < 0.05 (Fisher Exact with FDR multiple test correction). For GO analysis for biological processes of DE genes resulting from courtship conditioning terms were declared significant if they had a p-value of < 0.05 (Binomial test with Bonferroni correction). Results are displayed in ‘hierarchical view’, which groups similar terms together under the most enriched term to remove redundancy (Mi *et al.* 2017). Further functional analysis of the individual genes associated with each enriched term was provided by FlyBase (Gramates *et al.* 2017).

### Network analysis

Interaction network was generated using the GeneMANIA app in Cytoscape 3.4.0 (Montojo *et al.* 2014; Su *et al.* 2014). The network was generated using the following annotated networks: (1) physical interactions - biogrid small scale studies, (2) genetic interactions – biogrid small scale studies, and (3) predicted. No related genes were integrated into the network. Nodes were color annotated using the Cytoscape enhancedGraphics app (Morris *et al.* 2014). Each node was annotated based on association with relevant gene ontology terms.

### Data Availability

Figure S1 contains PCA results of MB and WH RNA sequencing data. Figure S2 contains comparison of DE results between DESeq2 and NOISeq. Table S1 contains read alignment and count data. Table S2 contains DE analysis results for MB specificity. Table S3 contains GO results for DE MB enriched or depleted genes. Table S4 contains DE analysis results for MB and WH specific samples during a time course of LTM. Table S5 contains the results of k-means clustering of DE genes during LTM formation. Table S6 contains GO results for clusters of DE genes identified during LTM formation. Gene expression data are available at GEO with the accession number: GSE115718. Supplemental material available at Figshare: https://doi.org/10.25387/g3.7005842.

## Results

### MB-unc84 males display normal long-term courtship memory

**Figure 1:**
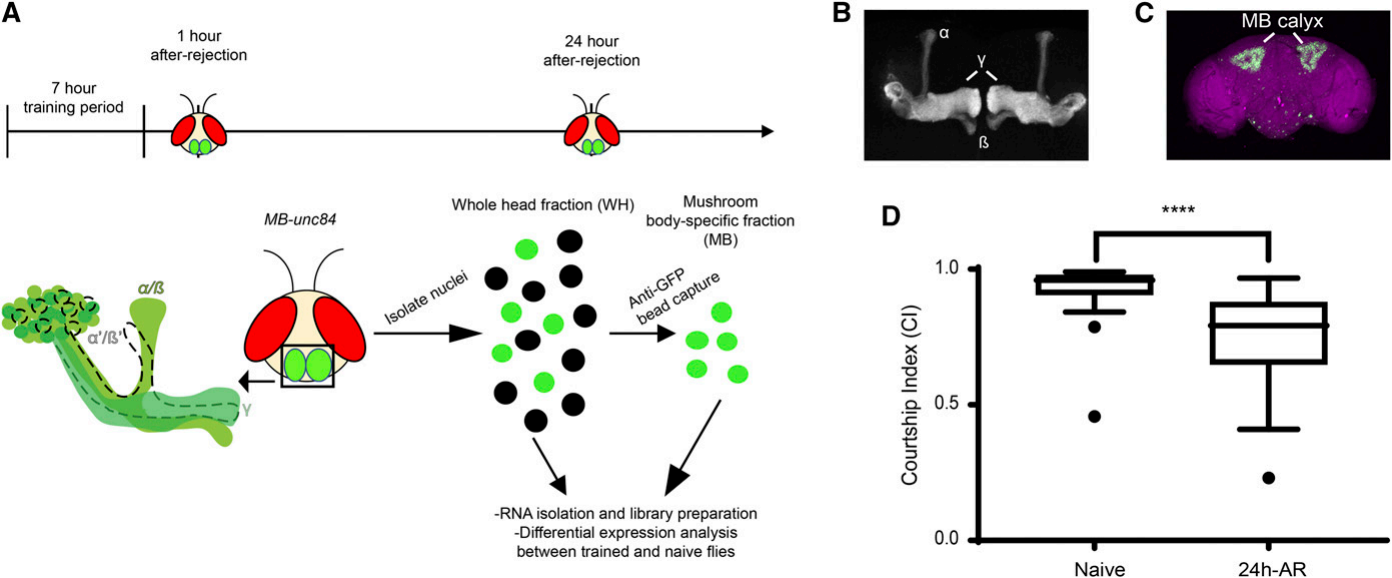
Schematic of the experimental design and validation of courtship conditioning to induce LTM. A) Long-term memory (LTM) was induced in flies using a previously established seven-hour courtship conditioning protocol (Koemans *et al.* 2017). Following training, *MB-unc84* flies expressing unc84::GFP in the α/β and γ lobes of the MB were collected for INTACT and downstream transcriptome analysis at two time points: one hour and 24 hr after-rejection (AR). RNA was then isolated from the WH and MB-fractions, cDNA libraries prepared, and next-generation sequencing performed. B) Confocal projection of the whole brain showing the expression of *UAS-unc84*::*GFP* using *R14H06-GAL4*. Only a few nuclei outside of the MB calyx are labeled with GFP. MB calyx is indicated. Counterstaining was performed using the nc82 antibody against *Bruchpilot*. C) Confocal projection showing expression of *UAS-mCD8*::*GFP* with *R14H06-GAL4* in the α/β and γ lobes of the MB. D) To provide evidence of normal memory in *MB-unc84*, a subset of flies were collected in parallel with flies used for transcriptome analysis and tested in the courtship conditioning assay. MB-unc84 flies show a decrease in courtship index (CI) at 24h-AR compared to Naïve flies, suggesting normal memory function in this genotype (n = 23 and n =29, respectively, for naïve and trained flies; **** *P* < 0.001 Mann-Whitney *U*-test).

The aim of this study was to identify MB-specific transcript level changes that occur after the acquisition of long-term courtship memory. To achieve this, we used INTACT (Deal and Henikoff 2010; Steiner *et al.* 2012; Henry *et al.* 2012) to isolate MB nuclei from fly heads, 1 h and 24 h after courtship conditioning (Figure 1A). We adapted a previously described INTACT protocol that employed a *UAS-unc84*::*GFP* transgene (Henry *et al.* 2012). Unc-84 is a *Caenorhabditis elegans* nuclear envelope protein, and when coupled with GFP, Unc84::GFP labeled nuclei can be immunoprecipitated from nuclear preparations derived from frozen tissue using an anti-GFP antibody. To drive expression of *UAS-unc84*::*GFP* in the MB, we used the *R14H06-GAL4* driver line from the Janelia flylight collection (Jenett *et al.* 2012). This driver is highly specific for the α/β and γ neurons of the mushroom body (Figure 1B), which are required for many forms of memory, including courtship conditioning (Montague and Baker 2016; Zhao *et al.* 2018; Koemans *et al.* 2017). *R14H06-Gal4* has a higher specificity than many classic MB Gal4 lines, which often have broad expression in the brain (Aso *et al.* 2009). The expression domain of *R14H06-Gal4* is available at (http://flweb.janelia.org/cgi-bin/flew.cgi) and provided here (Figure 1B-C). We generated flies that were heterozygous for both the *UAS-unc84* transgene and the *R14H06-GAL4* driver, which are hereafter referred to as *MB-unc84*.

To induce long-term courtship memory, *MB-unc84* males were paired with an unreceptive mated female for seven hours. Flies for transcriptome analysis were flash frozen at 1 h and 24 h after this period of sexual rejection - 1h-after rejection (AR) and 24h-AR (Figure 1A). These time points were selected to capture both early and late stages after memory acquisition. We avoided sampling during the rejection period to avoid the direct effect of being paired with a female (Ellis and Carney 2011, 2010). A minimum of four biological replicates was obtained for each time point. In parallel with these collections, we tested a subset of *MB-unc84* flies to confirm the induction of normal long-term courtship memory in these cohorts. Indeed, at 24h-AR *MB-unc84* males showed a robust reduction in courtship behavior in comparison to naïve males (Figure 1D; *P* < 0.001 Mann-Whitney *U*-test). This observed courtship suppression in *MB-unc84* flies was in line with expected values from the literature (McBride *et al.* 1999; Koemans *et al.* 2017; Keleman *et al.* 2007), demonstrating that *UAS-unc84*::*GFP* expression in the MB does not interfere with normal courtship memory.

### INTACT yields high-quality MB-enriched RNA

**Figure 2:**
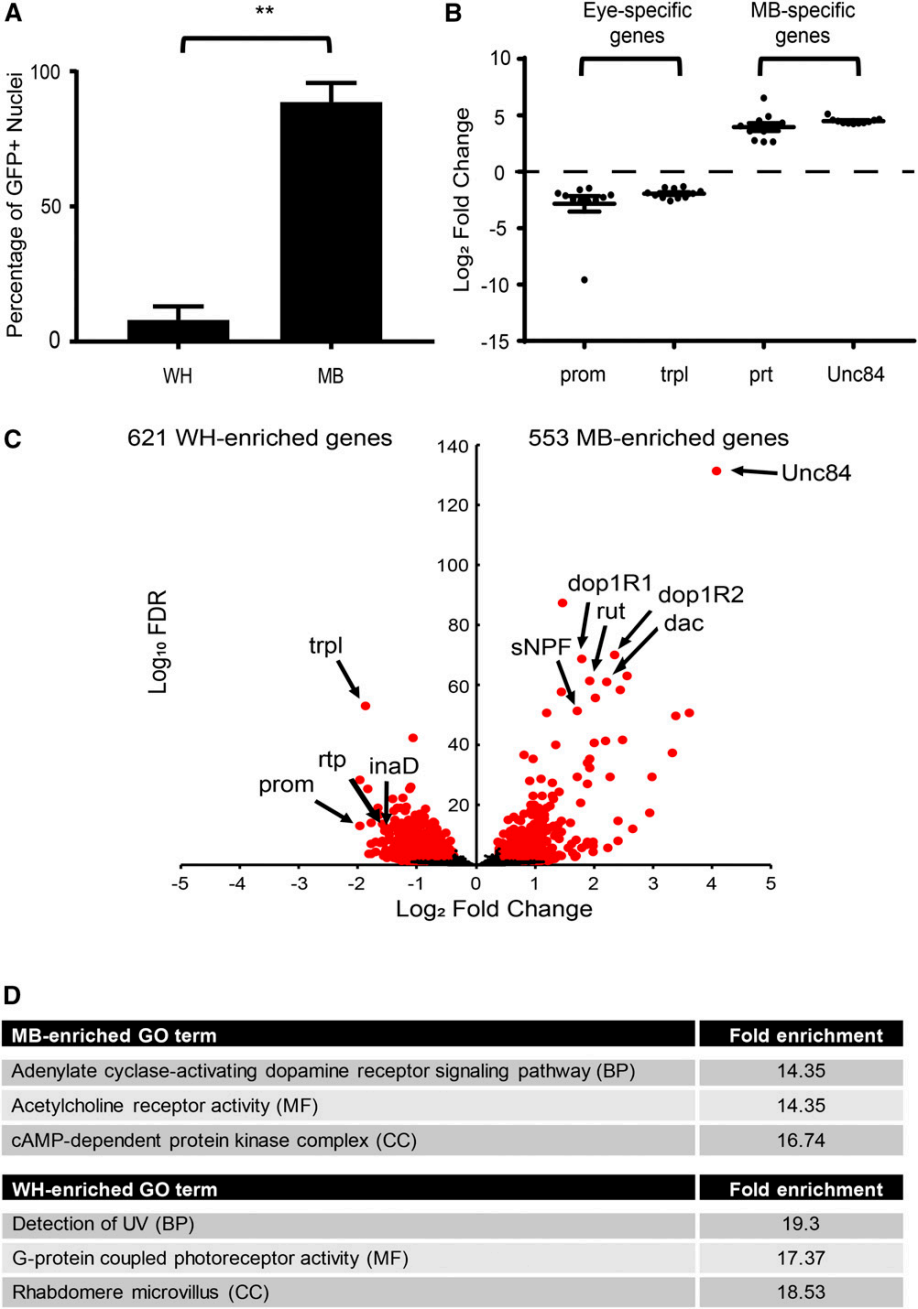
INTACT yields high-quality MB-enriched RNA. A) Graph showing the average percentage (± SD) of GFP positive nuclei in WH and MB samples obtained using INTACT. The percentage of GFP positive nuclei was determined by counting total nuclei labeled with DRAQ5 (** *P* < 0.01, Student’s *t*-test, n = 3). B) Dot plot showing log2 fold changes for a selection of genes with specific expression in the eye (*prom*, *trpl*), and the mushroom body (MB) (*prt*), as well as the nuclear tag *unc84*. Fold changes were calculated for n = 11 biologically paired WH and MB samples (See Table S1 for details of these samples). The mean and standard error of the mean are indicated. C) Volcano plot showing the results of differential expression (DE) analysis comparing all MB samples (n = 12) to all WH samples (n = 12) (Table S2). 553 and 621 DE genes were significantly enriched in MB and WH, respectively (q < 0.05, fold difference > 1.3). A selection of genes previously known to be enriched in the MB (*rut*, *dac*, *sNPF*, *Dop1R1*, *Dop1R2*) and optical lobe (*trpl*, *prom*, *rtp*, *inaD*), as well as *unc84*, are highlighted. D) Gene ontology (GO) enrichment analysis was performed for MB-enriched and WH-enriched genes. The most enriched GO terms for biological processes (BP), molecular functions (MF) and cellular components (CC) are displayed for both MB and WH-enriched genes (FDR corrected p-value < 0.05, Fisher exact test, minimum four genes, Table S3).

To provide evidence that our approach could obtain nuclei specific to the MB, we used fluorescent microscopy to measure the proportion of GFP-positive nuclei present in whole head (WH) extracts, compared to INTACT MB samples. WH nuclei obtained from *MB-unc84* flies contained 8% GFP positive nuclei (Figure 2A). Note that this is likely an overestimation, as we only analyzed fields of view containing GFP-positive nuclei, which were not present throughout the slide. After immunoprecipitation of nuclei from WH extracts using anti-GFP bound beads, about 90% of nuclei were GFP-positive, indicating a high level of specificity of our INTACT protocol (Figure 2A).

Next, INTACT was used to extract MB nuclei from *MB-unc84* heads at 1h-AR and 24h-AR, as well as from naïve flies matched for age and time-of-day. For each MB sample, we also obtained RNA from nuclei present in the biologically paired WH input for comparison. After verification of RNA quality, sequencing libraries were prepared from both WH and MB samples. Completed libraries were sequenced and reads were aligned to the *D. melanogaster* genome. Samples that had >10 million genic counts were included for downstream analysis, resulting in a total of 12 MB samples (four naïve, four 1h-AR, four 24h-AR) and 12 WH samples (five naïve, three 1h-AR, four 24h-AR) (Table S1).

To confirm consistent enrichment in MB samples we examined gene expression differences between WH and MB samples. DESeq2 was used to normalize gene counts between all MB and WH samples and genes with less than 50 counts across all samples were removed, leaving a total of 11941 genes with sufficient coverage. Log_2_ fold changes were then calculated using normalized counts for biologically paired WH and MB samples. For two samples we did not obtain a true biological pair due to failure during sample preparation, resulting in n = 11 samples that were used in this analysis (see Table S1 for details of read depth and biological pairing). As expected, eye-specific genes like *prom* and *trpl* were underrepresented in MB-samples, while MB-enriched genes, such as *prt* and *unc84* were overrepresented in the MB samples (Figure 2B). Notably, *unc84* expression was highly enriched and highly consistent across all biological replicates suggesting a high degree of consistency in MB-enrichment after INTACT.

To provide further evidence that the nuclei we isolated displayed MB-specific gene expression profiles we performed differential expression analysis between all MB (n = 12) and WH samples (n = 12). We identified 553 and 621 genes (q < 0.05, fold difference > 1.3) that were significantly enriched in either MB or WH samples, respectively (Figure 2C; complete list in Table S2). Many known MB-expressed genes, including *rut*, *dnc*, *prt*, *ey*, *toy*, and *dac* were among the most differentially expressed MB-enriched genes (Livingstone *et al.* 1984; Noveen *et al.* 2000; Brooks *et al.* 2011; Kurusu *et al.* 2000). In contrast, several eye-specific genes, such as *prom*, *trpl*, *inaD*, and *rtp*, were among the most differentially expressed WH-enriched genes (Figure 2C). Additionally, we compared MB-enriched genes to cell surface receptors that were found to be characteristically expressed in α/β and γ KC’s when compared to MBONs (Crocker *et al.* 2016). Indeed, many of these receptors were also found to be enriched in our dataset including: *Dop1R2*, *Dop1R1*, *Dop2R*, *5-HT1B*, *Oamb*, *Octβ1R*, *sNPF*, *GluRIB*, *Ir68a*, *CCKLR-17D1*, *CCKLR-17D3*, *GluRIB*, *and mAChR-A* (Table S2). Finally, we examined gene ontology (GO) terms enriched for biological processes (BP), molecular functions (MF), as well as cellular components (CC), among our lists of MB-enriched and WH-enriched genes (Figure 2D, Table S3). The most enriched GO terms for MB-enriched genes were “cAMP-dependent protein kinase complex” (CC) and “adenylate cyclase-activating dopamine receptor signaling pathway” (BP) (Figure 2D), which fits with the known importance of dopaminergic modulatory neurons and cAMP signaling in memory formation in the MB (Aso *et al.* 2014; Keleman *et al.* 2012; Blum *et al.* 2009; Zhang *et al.* 2015). The most enriched GO term for MF was “acetylcholine receptor activity”, consistent with previous studies which showed that MB KC’s are cholinergic and receive input from cholinergic olfactory projection neurons (Barnstedt *et al.* 2016; Crocker *et al.* 2016; Gu 2006). In contrast, the most enriched GO terms for the WH enriched genes were all related to eye function, including “detection of UV” (BP), “G-protein coupled photoreceptor activity” (MF) and “rhabdomere microvillus” (CC) (Figure 2D). Taken together, analysis of genes that are differentially expressed between WH and MB samples revealed a pattern of gene expression that is highly consistent with an effective MB enrichment.

### Gene expression changes after memory acquisition

**Figure 3:**
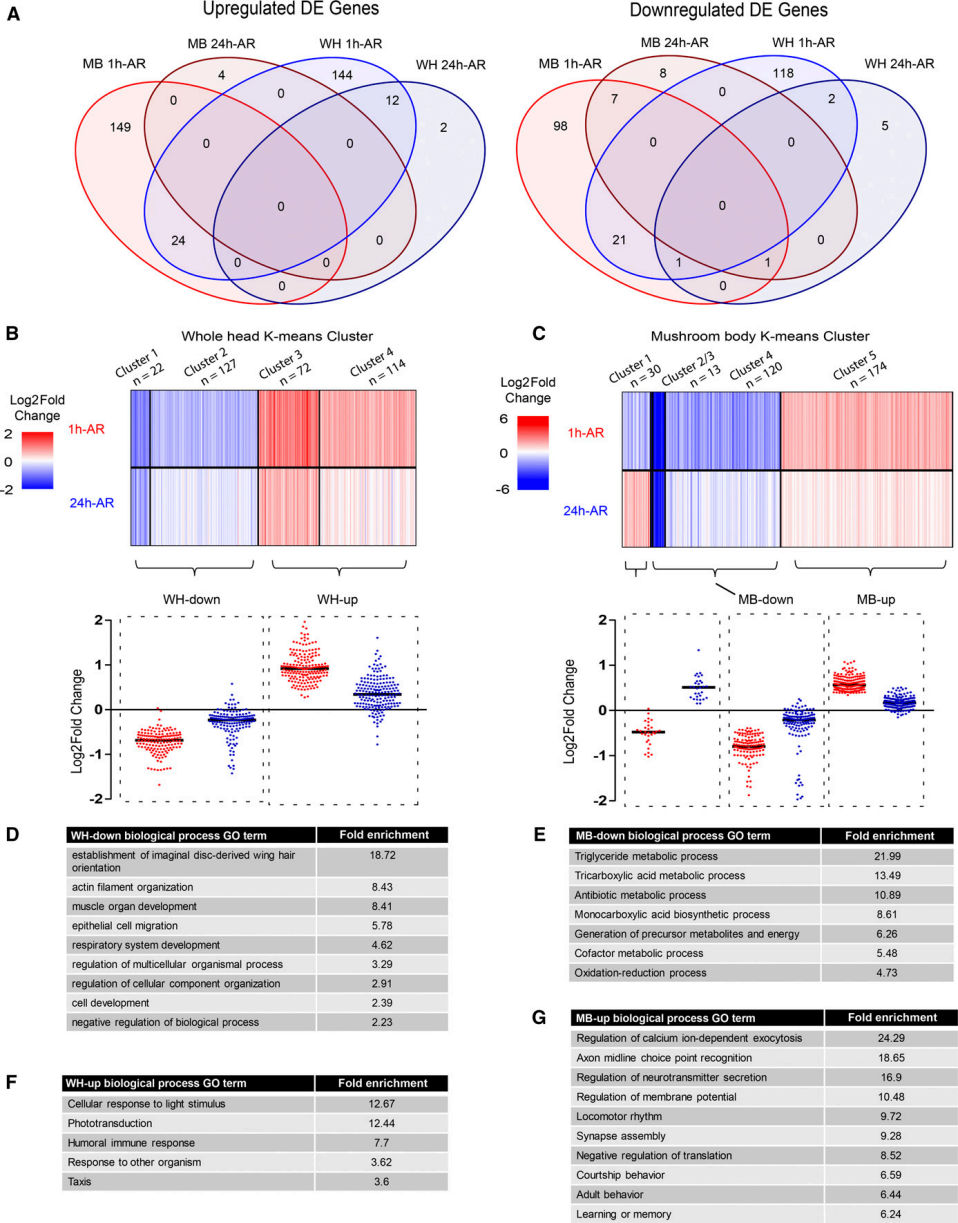
Differential expression and clustering analysis of MB and WH RNA sequencing. A) Venn diagram showing overlap between MB and WH differentially expressed (DE) genes (q < 0.05, fold difference 1.3 up or down) for both upregulated and downregulated genes (File S4). (B-C) Cluster analysis of WH and MB DE genes identified by DESeq2 (q < 0.05, fold difference 1.3 up or down). Log_2_ fold change data were obtained for significant DE genes at both one hour, as well as 24-hour time points and clustered using *k*-means. Heatmap shows the individual log_2_ fold changes for each gene. Dot plot shows log_2_ fold changes for genes with similar expression trends (File S5). (B) Four clusters were identified for WH DE genes, with two distinct trends. Cluster 1 and 2 were downregulated at both time-points (WH-down) and cluster 3 and 4 were upregulated at both time-points (WH-up). C) Five clusters were identified for MB DE genes with three distinct trends. Cluster 1 was downregulated 1h-AR and upregulated 24h-AR. Cluster 2, 3 and 4 were downregulated at both time-points (MB-down). Cluster 5 was upregulated at both time-points (MB-up). (D-G) GO enrichment analysis for biological processes using PANTHER (*P* < 0.05, Binomial test with Bonferroni correction, minimum 5 genes, sorted by hierarchical view). The top GO terms, heading each GO hierarchical cluster are displayed, sorted by fold enrichment (Table S6), for (D) WH-down, (E) MB-down, (F) WH-up, (G) MB-up.

Next, we used DESeq2 to identify genes that were differentially expressed (DE) in response to courtship conditioning by comparing 1h-AR and 24h-AR to naïve flies. DE analysis was performed on normalized counts for both WH and MB samples after genes with less than 50 mean counts across all samples were removed, which left 8730 and 8561 genes, respectively, with sufficient coverage. For both WH and MB samples we observed more DE genes at 1h-AR than at 24h-AR (n = 322/23, n = 302/20, for WH and MB sample 1h-AR/24h-AR, respectively). There was some overlap in DE genes between 1h-AR and 24h-AR, and between WH and MB samples, however, most DE genes identified in WH and MB samples were different (Figure 3A). To investigate trends in gene expression after courtship conditioning we compiled a list of all DE genes that were differentially expressed in at least one of the three pairwise comparisons: 1h-AR *vs.* naïve, 24h-AR *vs.* naïve, and 1h-AR *vs.* 24h-AR (Table S4). This led to the identification of 332 and 342 DE genes for WH and MB samples, respectively. For each tissue, we performed *k*-means clustering on log2 fold changes at 1h-AR and 24h-AR (Figure 3B and 3C, Table S5). In WH samples, four clusters were identified with two distinct trends: cluster 1 and 2 (n = 22 and 127) contained genes that were downregulated at 1h-AR and either reduced or not changed at 24h-AR (WH-down, Figure 3B and Table S5). Cluster 3 and 4 (n = 72 and 114) contained genes that were upregulated at 1h-AR and either less upregulated or not changed at 24h-AR (WH-up - Figure 3B and Table S5). For MB samples *k*-means clustering revealed five clusters with three distinct expression trends. Cluster 1 (n = 30) contained genes that were downregulated at 1h-AR and upregulated 24h-AR. Clusters 2, 3, and 4 (n = 2, 13 and 120, respectively) contained genes that were downregulated at 1h-AR and either downregulated or not changed at 24h-AR (MB-down - Figure 3C and Table S5). Cluster 5 (n = 174) contained genes that were upregulated at 1h-AR and either upregulated or not changed at 24h-AR (MB-up - Figure 3C and Table S5). This clustering allowed us to identify gene groups with similar expression trends and emphasized the relatively strong effect of sexual rejection at 1h-AR.

### Courtship conditioning is associated with MB-specific downregulation of metabolic genes

To investigate the functions of genes that are differentially expressed in response to courtship conditioning, we first performed GO enrichment analysis for gene clusters with similar expression trends. For WH-down genes (n = 149, Figure 3B) we observed, almost exclusively, enrichment of GO terms related to development, for example, “metamorphosis”, “cell differentiation”, and “cell migration” (Figure 3D and Table S6). For MB-down genes (n = 135, Figure 3C) we observed enrichment only of GO terms related to metabolism (Figure 3E and Table S6). In fact, over half (n = 73) of the MB-specific downregulated genes are annotated with the term “metabolic processes” (Table S6). Notably, there was no overlap in enriched GO terms between WH-down and MB-down genes. The highly specific effect of courtship conditioning on the regulation of metabolic genes in the MB is very interesting as metabolic changes are known to be important for the formation of LTM and in response to synaptic activity (Bas-Orth *et al.* 2017; Segarra‐Mondejar *et al.* 2018; Goyal *et al.* 2014; Plaçais *et al.* 2017; Tadi *et al.* 2015). We see MB-specific downregulation of genes encoding mitochondrial proteins involved in the Krebs cycle (Mdh1, Acon, Ldh, ScsβA) and the electron transport chain (blw, ATPsynβ, ATPsynγ, ND-51) (Table S5). This downregulation of genes involved in oxidative glucose metabolism suggests a shift toward aerobic glycolysis, known as the Warburg effect, where cells favor non-oxidative glucose metabolism despite the presence of oxygen (Chen *et al.* 2015). This type of metabolism is seen in mammalian neurons in response to synaptic activity and LTM formation (Bas-Orth *et al.* 2017; Segarra‐Mondejar *et al.* 2018; Tadi *et al.* 2015). Aerobic glycolysis may serve to protect neurons against oxidative damage and has been suggested as a mechanism to provide precursor molecules that are required for synaptogenesis (Bas-Orth *et al.* 2017; Segarra‐Mondejar *et al.* 2018; Goyal *et al.* 2014; Tadi *et al.* 2015).

### Courtship conditioning is associated with MB-specific upregulation of synaptic proteins and learning and memory pathways

**Figure 4:**
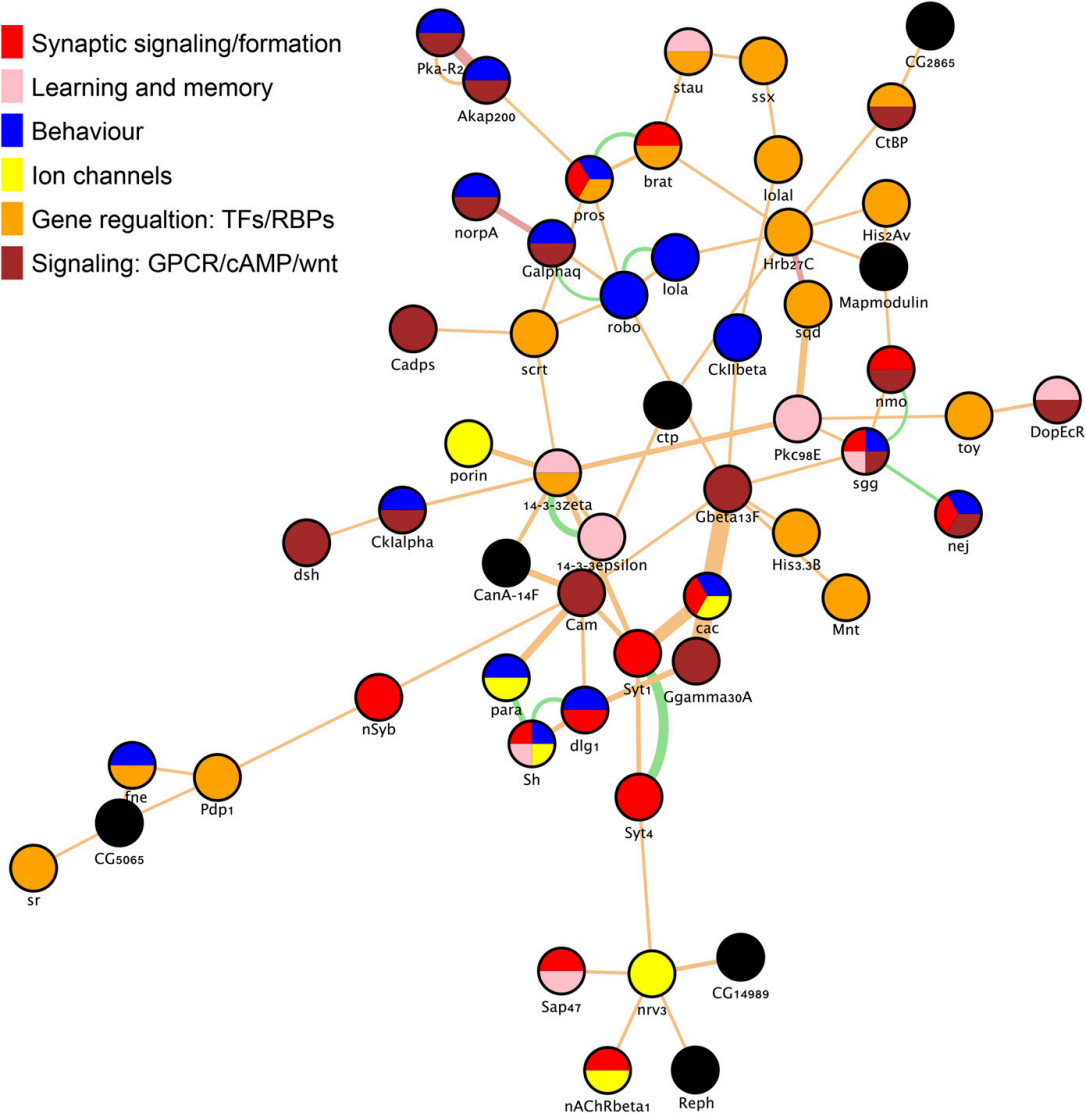
Network analysis of genes that are upregulated in the MB in response to courtship conditioning. Of 178 genes in the MB-up group, 54 form a single network based on a subset protein-protein and genetic interactions that are annotated in geneMANIA (see methods). Each node is color coded to represent selected gene ontology annotations.

For WH-up genes (n= 186, Figure 3B) all enriched GO terms were related to biological responses, such as “cellular response to light stimulus”, “humoral immune response”, “response to other organism”, and “taxis” (Figure 3F and Table S6). Indeed, 62 of the WH-up genes were annotated with the term “response to stimulus” (Table S6). GO terms related to biological response were also enriched for MB-up genes (n = 174, Figure 3C). There were 5 enriched GO terms common to WH-up and MB-up genes (“response to light stimulus”, “response to abiotic stimulus”, “response to stimulus”, “response to external stimulus”, and “taxis”) (Table S6). Yet the MB-up gene group showed many more enriched GO terms - 181 compared to 15 for WH-up - suggesting a high level of functional relatedness in this gene group. Using annotated protein-protein and genetic interactions, we identified a network of 54 MB-up genes (Figure 4). This network was comprised of genes encoding ion channels, transcription factors, RNA binding proteins, and genes with functional annotations related to synapse formation, synaptic signaling, behavior, and learning and memory (Figure 4). Interestingly, some of the most enriched GO categories that were unique for MB-up genes were related to synaptic plasticity (*e.g.*, “regulation of calcium ion-dependent exocytosis”), behavior (*e.g.*, “courtship behaviour”), and memory (“*e.g.* “learning or memory”) (Figure 3G). Taken together, these results suggest that MB-up genes encode a highly interactive group of proteins with biological relevance to learning and memory.

**Figure 5:**
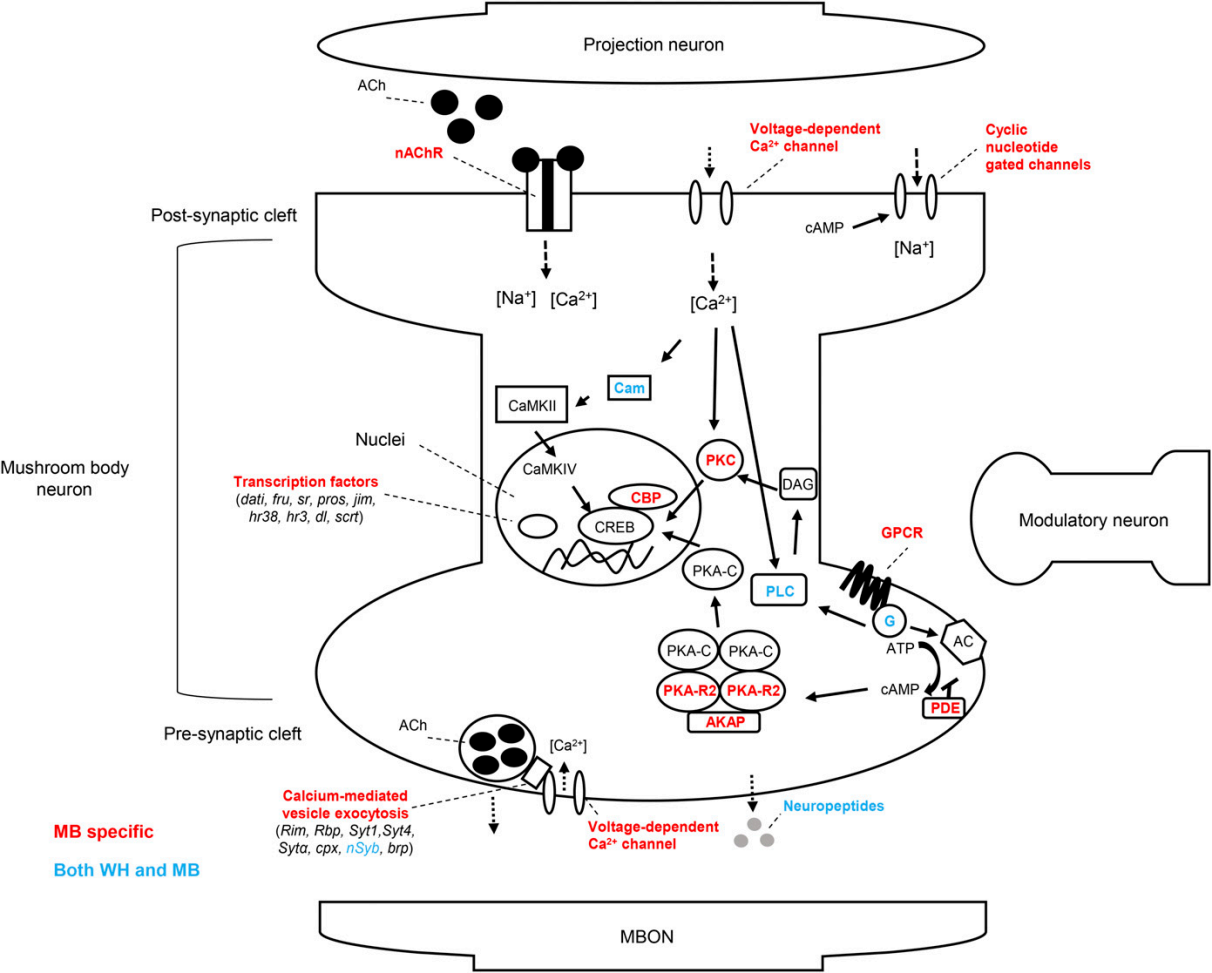
Schematic representation of molecular pathways underlying memory in the mushroom body. Manually curated diagram of memory-relevant molecular pathways in MB Kenyon cells which were identified to be differentially expressed in the MB-up gene group (shown in red). Calcium-dependent, cholinergic, and cAMP signaling pathways are among the molecular pathways represented. Additionally, genes encoding proteins involved in calcium-mediated presynaptic neurotransmitter release, as well as differentially expressed transcription factors are shown.

Next, we manually curated the MB-up gene group to illustrate how they may be represented in memory-relevant molecular pathways in MB KCs (Figure 5). During learning and memory formation KCs receive olfactory input from over 200 olfactory projection neurons (PNs) that synapse with the dendrites of the calyx (Caron *et al.* 2013). Olfactory signals are reinforced to form memories by sensory signals from modulatory dopaminergic neurons (DANs), which synapse at discrete locations along the axons of the MB lobes (Aso *et al.* 2014). In courtship conditioning, the primary olfactory signal is thought to be the pheromone cVA, which is deposited on females by males during mating (Everaerts *et al.* 2010). Courtship memory is formed when cVA is paired with sexual rejection, which is conveyed to the MB γ lobe via a specific class of DANs (Keleman *et al.* 2012). Long-term courtship memory is also dependent on the production of the hormone ecdysone, which can also act as an input signal to KCs (Ishimoto *et al.* 2009; Ishimoto *et al.* 2013). Olfactory PNs are cholinergic and are thought to stimulate KCs through activation of nicotinic acetylcholine receptors (nAChRs), which are ligand-gated channels that induce calcium influx into KCs (Christiansen *et al.* 2011; Yasuyama *et al.* 2002; Campusano *et al.* 2007). Calcium influx is required for downstream signaling associated with synaptic plasticity and memory formation (Lisman 1994). Among MB-up genes, we noted several genes involved in receiving olfactory signals and mediating downstream calcium dependent signaling (Figure 5). These included genes encoding three nAChR subunits (nAChRα1, nAChRα6, nAChRβ1), the acetylcholinesterase (Ace) involved in acetylcholine recycling, the voltage-gated calcium channel *Ca-β*, the calcium-activated signaling proteins PLC and PKC, and the calcium-binding messenger calmodulin (Cam) (Widmann *et al.* 2016; Pavot *et al.* 2015; Campusano *et al.* 2007). Many MB-up genes also encode proteins involved in receiving modulatory signals, and in the cAMP signaling pathway that is activated by these signals during memory formation (Figure 5). Notably, we identified MB-specific upregulation of four G-protein coupled receptors (GPCR). These included oamb, hec, and SIFaR, all known to be involved in male courtship behavior (Li *et al.* 2011; Sellami and Veenstra 2015; Zhou *et al.* 2012), and DopEcR, an atypical GPCR that responds to both dopamine and ecdysone, and is essential for cAMP signal activation during courtship memory (Ishimoto *et al.* 2013). We identified five MB-up genes encoding components of the heterotrimeric G-protein complex (Gαq, Gβ13F, Gγ30A, Gαo, Gγ1), which acts directly downstream GPCRs to induce adenylate cyclase activity and production of cAMP (Livingstone *et al.* 1984; Levin *et al.* 1992). Several downstream cAMP signaling components were also upregulated specifically in the MB, including regulatory subunits of protein kinase A (PKA-R2), the PKA anchoring protein (Akap200), cAMP-gated ion channels (*Ih*, *Cngl*), and the CREB-binding protein, nej, a histone acetyltransferase that is thought to be involved in LTM-associated gene expression (Alarcón *et al.* 2004; Hirano *et al.* 2016). Thus, many MB-up genes are directly related to receiving and processing the signals that induce courtship memory.

KC axons provide presynaptic output to 21 MBONs (Aso *et al.* 2014). Several MB-up genes encode proteins involved in calcium-mediated presynaptic neurotransmitter release, including the synaptic vesicle docking proteins RIM and RBP, the synaptotagmins (Syt1, Syt4, Sytα), components of the SNARE complex (cpx and nSyb), the presynaptic calcium channel cacophony, and the active zone marker brp (Figure 5) (Deitcher *et al.* 1998; Kittel and Heckmann 2016; Huntwork and Littleton 2007; Liu *et al.* 2011; Knapek *et al.* 2011). We also observed upregulation of two neuropeptides, Nplp2, and sNPF. sNPF is has been shown to act synergistically with ACh in communicating to MBONs in the context of olfactory memory formation (Barnstedt *et al.* 2016). Thus, many MB-up genes are involved in pre-synaptic neurotransmission and it can be inferred that these genes may play a role in transmitting memory signals to MBONs (Figure 5).

Finally, we also observed upregulation of many genes encoding transcription factors and RNA binding proteins. RNA binding proteins like stau and Orb2 are thought to be involved in LTM formation through localized regulation of translation at synapses (Dubnau *et al.* 2003; Khan *et al.* 2015). Some of the transcription factors in the MB-up group have known roles in courtship behavior, such as dati, fru and pros (Grosjean *et al.* 2007; Schinaman *et al.* 2014; Manoli *et al.* 2005). Interestingly, we identified MB-specific upregulation of sr and Hr38, which are transcription factors that have been proposed as markers of neuron activation in insects (Fujita *et al.* 2013; Lutz and Robinson 2013; Chen *et al.* 2016).

## Discussion

Understanding transcriptional changes that are required in neurons to mediate LTM is an important challenge in neuroscience. Many studies have identified gene expression changes after memory acquisition in *Drosophila* (Winbush *et al.* 2012; Bozler *et al.* 2017; Dubnau *et al.* 2003; Crocker *et al.* 2016) and this approach has been used to identify new genes involved in memory formation (Bozler *et al.* 2017; Dubnau *et al.* 2003; Crocker *et al.* 2016). However, we still understand very little about the spatial and temporal requirement for transcription in LTM. When are critical memory genes activated and in which neurons? Here, we used MB-specific transcriptional profiling to identify changes in transcript levels that occur in response to courtship conditioning, an ethological memory paradigm that is commonly used in *Drosophila*. This analysis revealed gene expression changes in established learning and memory pathways that occurred for the most part at 1 hr after courtship rejection, but not after 24 hr. Importantly, canonical memory related pathways were only differentially regulated in the MB and not in biologically paired WH samples. These results suggest that memory related biological processes are transiently upregulated in the MB after memory acquisition, illustrating the importance of sampling time, as well as cell type, in the identification of biologically relevant gene regulation in LTM and offer a valuable list of candidate genes for further investigation.

In our study, we compared males that experienced sexual rejection to naïve socially isolated males. Although samples were collected at least one hour after exposure to a female fly, it is impossible to conclusively differentiate between transcript level changes that occur because of sexual rejection - and long-term memory formation - and changes that might happen in response to any social interaction. Previous studies have looked at gene expression changes that occur in whole heads in response to courtship, male-male interactions, and mating (Ellis and Carney 2011, 2010). As could be expected, in WH samples we do observe a significant overlap with those studies (36 genes, 1.5-fold enrichment, *P* < 0.001). This suggests that some gene expression changes in whole heads represent general responses to social interactions. In contrast, we see no significant overlap between genes identified in these social interaction studies and genes that we observe to be changed in the MB after courtship conditioning (6 genes, 0.26-fold enrichment, *P* > 0.05). This is consistent with the fact that the MB is not required for normal male-female interactions like courtship behavior or mating as MB-ablated flies reproduce normally and even show normal learning in response to sexual rejection (de Belle and Heisenberg 1994; McBride *et al.* 1999). Therefore, it is reasonable to suggest that MB-specific gene expression changes we observed are likely related to memory acquisition.

One major limitation of this study is that we have not functionally or technically validated the DE genes. As with any genomic dataset, there will be false positives. However, several factors suggest that many of the DE genes identified here have a high potential to be true positives and that our overall conclusions are not affected by the presence of false positives. First, we have used several biological replicates as well as a sophisticated software package, DESeq2, which performs among the highest in several measures of DE analysis quality (true positive rate, accuracy, positive predictive value, accuracy) when compared to other available software tools in an independent study (Costa-Silva *et al.* 2017). Second, we have compared results obtained with DESeq2 to NOISeq, another DE analysis program with high ranking quality measures (Tarazona *et al.* 2015; Costa-Silva *et al.* 2017). Although the list of DE genes does differ between the two programs, there is a high degree of overlap in the specific genes, resulting in a very similar enrichment of GO terms for genes that are differentially expressed 1h-AR (Figure S2). Third, some memory induced transcript changes observed here have been seen in other studies, providing indirect technical validation of our dataset. For example, *stau* was shown to be upregulated in whole heads after olfactory conditioning (Dubnau *et al.* 2003). We also see a significant overlap of genes upregulated 24 hr after rejection in Winbush *et al.* (3 genes, 50-fold enrichment, *P* < 0.001), where WH transcript levels were also measured 24 hr after courtship memory acquisition (Winbush *et al.* 2012). Finally, genes that are upregulated in the MB after memory acquisition (from both DESeq2 and NOIseq) show a remarkable correlation with known memory pathways. From post-synaptic receptors, to signaling pathways, to presynaptic neurotransmitter release mechanisms, nearly all known aspects of memory related synaptic plasticity are accounted for (Figures 3-5). In the MB-up group, there are 13 established learning and memory genes (Table S6), thus, functional validation of our dataset is available from the literature.

In general, other *Drosophila* memory transcriptome studies have not observed such a profound effect on known memory related genes and pathways (Crocker *et al.* 2016; Dubnau *et al.* 2003; Bozler *et al.* 2017; Winbush *et al.* 2012). This is likely due to both the sampling time and cell type we investigated. Certainly, memory specific transcriptional signals would be diluted in whole head analysis (Winbush *et al.* 2012; Bozler *et al.* 2017; Dubnau *et al.* 2003). In comparison to other MB specific transcriptome analyses, we do not see overlap in DE genes identified by Widmer *et al.* who profiled MB gene expression for 72 h after olfactory memory acquisition using DamID (Widmer *et al.* 2018). The lack of overlap may be due to differences in the Gal4 driver used targeting a different subset of MB neurons, the different memory paradigm (appetitive olfactory conditioning), or the differences in sampling time. Whereas we have obtained a snapshot view of mRNA transcript levels at two specific time points, Widmer *et al.* collected cumulative changes occurring over four 12-hour periods using the powerful DAM-ID method, which tracks transcription by indirectly measuring the association of polII with DNA. Crocker *et al.* used cell-specific patch clamping to investigate gene expression from MB neurons labeled by the c739-Gal4 and NP1131-Gal4 drivers, 30 min after memory acquisition. However, they identified very few differentially expressed genes in these neurons, which as they explain, is likely due to pooling of many samples that were conditioned with different odors (Crocker *et al.* 2016). The fact that we observed many expected memory genes and pathways to be induced in the MB suggests that we have serendipitously captured a critical time point for gene regulation in the formation of long-term courtship memory. Additionally, it stands to reason that the genes we identified that have not been previously associated with known memory pathways may represent novel mechanisms, which could be further investigated.

Many studies in mouse have profiled transcriptional changes in the hippocampus in response to fear conditioning and other memory paradigms (Zovkic *et al.* 2014; Vogel-Ciernia *et al.* 2013; Tadi *et al.* 2015). Consistent with our observations, these studies show many gene expression changes 30 min after memory acquisition, and not at later time points (Peixoto *et al.* 2015). In general, however, these studies do not identify widespread differential expression of canonical learning and memory pathways as we do in the fly MB (Zovkic *et al.* 2014; Vogel-Ciernia *et al.* 2013; Tadi *et al.* 2015). In mouse, across many different studies, fear conditioning consistently invokes strong activation of immediate early genes such as c-Fos, which are known to be induced in response to neuron firing (Mayford and Reijmers 2016). In insects, neuron activity induced genes have been more elusive, however, two genes, *Hr38* and *sr*, are consistently upregulated in response to a variety of neuronal activation stimuli in flies and other insects (Fujita *et al.* 2013; Lutz and Robinson 2013; Chen *et al.* 2016; Adhikari *et al.* 2018). It is very interesting that we observe these two genes to be specifically activated in the MB in response to sexual rejection. No other *Drosophila* memory-related transcriptome study has identified induction of these genes (Bozler *et al.* 2017; Winbush *et al.* 2012; Dubnau *et al.* 2003; Crocker *et al.* 2016; Widmer *et al.* 2018), except for Crocker *et al.* who did identify *Hr38* induction in the MB α/β cells at 30 min after memory acquisition, albeit with a borderline q-value (0.058) (Crocker *et al.* 2016). This suggests that our MB-specific analysis, coupled with an appropriate sampling time, has revealed a parallel mechanism to mammals that has not previously been observed in flies, where the induction of neuron activity induced genes is observed following memory acquisition.

In the future, it will be important to further refine the cell types and sampling times to fully understand transcriptional dynamics associated with memory formation. Indeed, even by focusing on less than 2000 MB cells, the actual circuit involved in the formation and long-term maintenance of the memory is likely composed of far fewer cells. The specific circuits that are required for courtship memory and other memory forms are being elucidated rapidly (Montague and Baker 2016; Zhao *et al.* 2018) and tools are now becoming available to label these cell populations for genomic analysis (Southall *et al.* 2013; Henry *et al.* 2012; Crocker *et al.* 2016). It is likely that further focus on more discrete cell populations will be required to fully understand gene activation in LTM.
